# Editorial: Animal models for pharmacological investigation of treatments and diagnostics for diseases

**DOI:** 10.3389/fcell.2022.1023512

**Published:** 2022-10-03

**Authors:** Natália Martins Feitosa, Rafael Henrique Nóbrega

**Affiliations:** ^1^ Integrated Laboratory of Translational Biosciences (LIBT), Institute of Biodiversity and Sustainability (NUPEM), Federal University of Rio de Janeiro, Macaé, Rio de Janeiro, Brazil; ^2^ Reproductive and Molecular Biology Group, Department of Structural and Functional Biology, Institute of Biosciences, São Paulo State University (UNESP), Botucatu, Brazil

**Keywords:** animal models, metabolism, endocrine system, reproduction, disease model, neuroprotective, drug therapy, new targets

In the last decades, interdisciplinary science has been shaping modern science to understand life, health, and disease (Erskine et al., 2013; Zhang et al., 2017; Mazzocchi, 2019). Along this path, animal models played a fundamental role in building knowledge from basic to applied science and diseases (Robinson et al., 2019; Mukherjee et al., 2022) including more recently in SARS-CoV-2 studies (Caldera-Crespo et al., 2022). *In vitro* and *in silico* studies are undoubtedly relevant, however, animal models in research are still essential and their use has been largely accepted in the scientific community. These debates led to the ethical improvement of animal welfare rules in many countries, which avoids unnecessary use of animals and animal cruelty (review Robinson et al., 2019). Therefore, much of the pharmacological investigation and other fields of science benefit from animal models.

**FIGURE 1:**
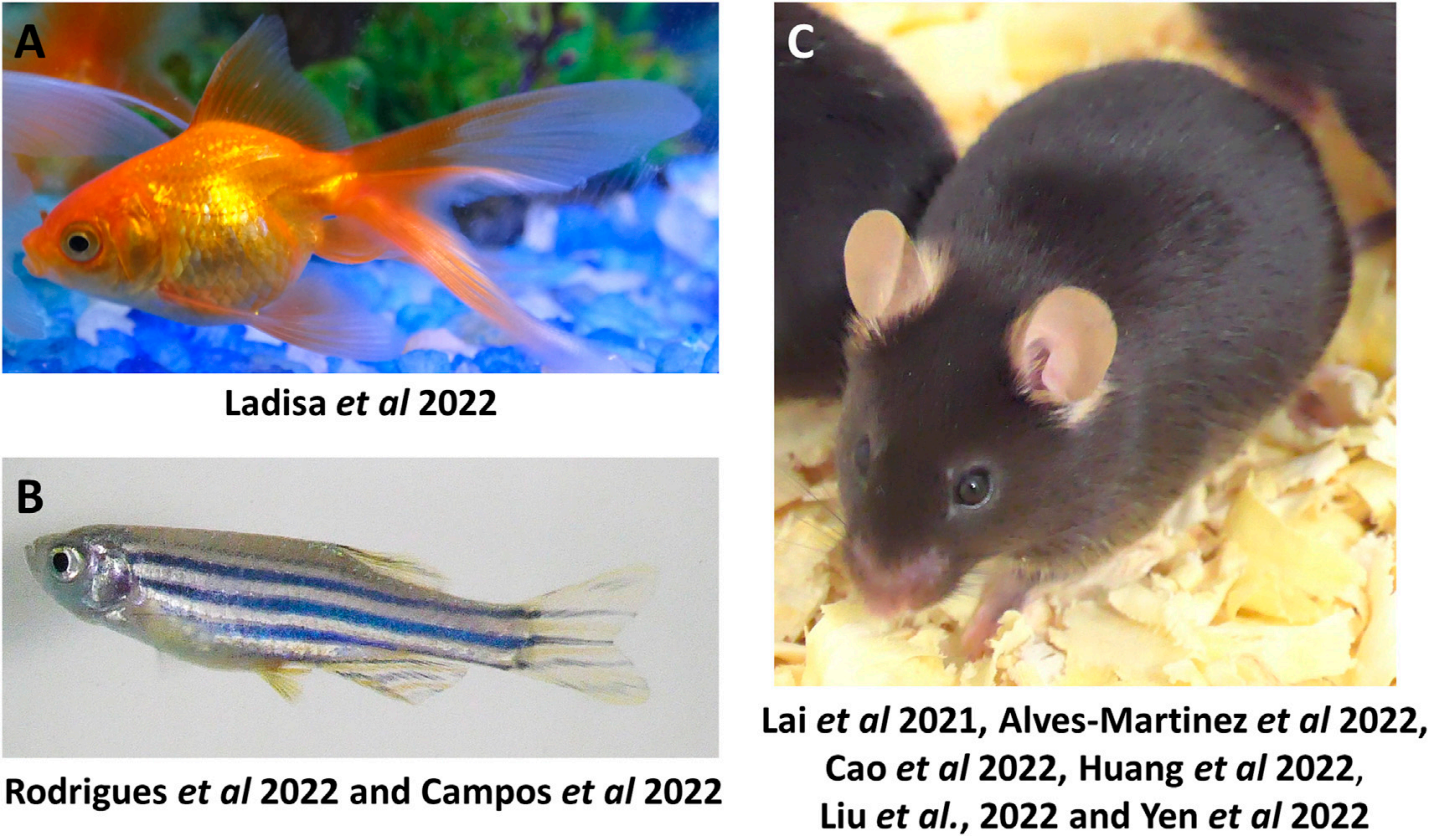
Animals used as experimental models in pharmacology research. **(A–C)** Representative pictures of the animal models and the respective research papers related to them. **(A)** goldfish (*Carassius auratus*), **(B)** zebrafish (*Danio rerio*); and **(C)** mouse (*Mus musculus*). A C57BL/6J mouse picture is shown.—In some studies published in this Research Topic, the Balb-c (picture not shown) species was used.

In this Research Topic, mostly murine and fish models were used to introduce new studies to understand not only diseases but normal development and homeostasis (Figure 1). In the case of fish models, Ladisa et al. presented the goldfish as a great model to study energy attribution related to reproduction and growth, through metabolomics. More specifically, they demonstrated that changes in metabolism may affect the development and growth of gonads in oviparous fish. Gonadotropin Inhibitory Hormone (GnIH) would play a role in this regulation, including lipid yield as preparation for egg production. Although goldfish is a seasonal response animal, GnIH signaling might be a conserved pathway among vertebrates. Increased GnIH levels in mice for example affect adiposity and reproduction (review Bédécarrats et al., 2022). In the light of hormone crosstalk with metabolism and gonad maturation, Rodrigues et al. showed that thyroid hormones had an effect on zebrafish spermatogenesis. The impairment of thyroid hormone production and activity through methimazole directly impacted sperm production and genes related to the control of spermatogenesis Ladisa et al. and Rodrigues et al. studies demonstrated that the endocrine system, reproduction, and metabolism are intimately related. The understanding of the connections among those pathways is fundamental to assessing the impact of endocrine disruptors in the organism of vertebrates.

A classic substance for human control of hypercholesterolemia, simvastatin, was revisited for drug safety using zebrafish embryos and chicken cell culture. Campos et al. group used the zebrafish embryos and chicken muscle culture as models to investigate the unwanted side effects of simvastatin on the muscles of human patients. The authors indicated that simvastatin could affect muscle formation and inhibit cell proliferation *in vivo* and *in vitro*. Campos et al. presented their results, provided a small review of the literature, and opened an important discussion on the use of simvastatin in clinics. They highlighted that when dealing with muscle degenerative diseases, simvastatin reduction of cell proliferation in muscle regeneration should be considered.

The genes and proteins do not only act in the normal development and differentiation of organs but can play a protective role in pathological events, such as kidney acute or chronic injury. Acute kidney injury (AKI) is defined as a sudden decay in the capacity of glomerular kidney filtration (Han and Thomas Lee, 2019). AKI can evolve into chronic kidney disease and/or death of the patient and some of the causes include sepsis and renal ischemia-reperfusion injury (IRI) (Murugan and Kellum, 2011; Han and Thomas Lee, 2019). IRI may happen after the interruption of renal blood flow followed by subsequent reperfusion that leads to renal dysfunction (Han and Thomas Lee, 2019). In the literature, it has been described that the severity of the injuries has a sex-related trait. Males were more sensitive to renal IRI than females (Kang et al., 2014). The following studies used mice as the main animal model. Huang et al. studied renal IRI in mice and found a differential expression of androgen receptor (AR) and microRNA-21 (miR-21) expression. The AR and miR-21 could inhibit the expression of caspase-induced apoptosis and were more greatly expressed in females than males, and show a protective effect on renal IRI. The role of AR has been extensively studied in the literature related to sex differentiation and male maturation, but the role in other organs such as kidney still need elucidation. In chronic diseases such as diabetic nephropathy (DN), differently from AKI, rarely present histological changes, the parenchyma of the glomerulus is compromised and leads to the disorder’s progression (Akchurin, 2019). Cao et al. revealed that high glucose (HG) increased oxidative stress and podocyte cell death through increased activity of cyclin-dependent kinase 5 (CDK5). This enzyme has multiple functions in cell homeostasis including survival. The authors found that TFP5, a specific inhibitor of CDK5, was able to decrease inflammatory cytokines and oxidative stress protecting the kidney of the diabetic mice model. These data, together with cell culture and RNAseq analyses, emphasized two other Sirt1 and nerve growth factor (NGF) players that could prevent DN progression. Therefore, Cao et al. pointed out the importance of CDK5-NGF/Sirt1 regulating axis in DN and suggested the TFP5 for drug therapy.

Other research groups used an omic approach to investigate new target genes related to the metabolism of parasite infection (Liu et al.) or apnea-induced cardiac injury (Lai et al.). Both groups studied the pathogenesis of public health interest diseases and used mice as a model organism. Liu et al. focused on the liver transcriptomic maps of mice infected with the parasite that causes echinococcosis. This disorder can also be passed from dogs and foxes to humans by food and water intake contaminated with the parasite eggs (Kotwa et al., 2019). Liu et al. suggested several potential lncRNA-mRNA-miRNA axes during *Echinococcus multilocularis* infection that might be important for understanding the disease mechanisms. Long-non coding RNAs (lnRNAs), micro-RNAs (miRNAs), and ncRNAs reportedly play important roles in regulating chromatin remodeling, participating in mRNA transcription, modulating cell differentiation, apoptosis among other functions (Mercer et al., 2009; O’Brien et al., 2018). Lai et al. studied a new class of non-coding RNA, the circular-RNAs (cicRNAs) in the mice model with obstructive sleep apnea (OSA). OSA is a disorder that affects millions of people worldwide and is an important risk factor for cardiac morbidities (Lebkuchen et al., 2021). In this publication, they performed microarray and qPCR analyses, which showed two differentially expressed circRNAs and a circ-miRNA-mRNA regulation network, suggesting that these findings could unveil the pathophysiological mechanisms on OSA-associated cardiovascular disease. Both the studies of Liu et al. and Lai et al. reaffirm the importance of interdisciplinary work to elicit possible new therapeutic targets for diagnostic and treatment of diseases.

Interestingly, nutrients in the human diet such as caffeine and kefir have value in the possible treatment of disorders. Alves-Martinez et al. studied the benefits and the neuroprotective effect of caffeine in germinal matrix-intraventricular hemorrhage (GM-IVH) in mice. GM-IVH is a disease that is most frequently found intracranially in preterm infants. The authors presented multiple results suggesting that caffeine diminished brain complications related to GM-IVH. Furthermore, Yen et al. analyzed the properties of Kefir peptides (KP) on a mice model that mimics hemophilia. Hemophilic patients experience osteoporosis as a prevalent comorbidity. KP treatment could ameliorate the inflammatory levels of IL-6 and osteoclastogenesis. Their results suggest the use of KP as a complementary therapy for osteoporosis in hemophilic patients.

In summary, each study add knowledge about signaling pathways that protect or enhance the severity of diseases, leading to a more accurate choice of new targets for diagnostics and future therapies.
